# What’s my age again? Assessing the impact of stink bug egg mass age on host recognition by egg parasitoids *Trissolcus basalis* and *Trissolcus japonicus* (Hymenoptera: Scelionidae)

**DOI:** 10.3389/fphys.2025.1588946

**Published:** 2025-07-17

**Authors:** Anaïs Sion, Ivan Hiltpold, Marilyn Cléroux, François Verheggen, Diana la Forgia

**Affiliations:** ^1^ Chemical and Behavioral Ecology, Gembloux-Agro-Bio-Tech, TERRA, University of Liège, Liège, Belgium; ^2^ Entomology and Nematology, Plant Protection Strategic Research Division, Agroscope, Nyon, Switzerland; ^3^ Changins-Haute Ecole de Viticulture et Oenologie, Nyon, Switzerland

**Keywords:** biocontrol, chemotaxis, host recognition, short-range cues, trophic cascade, volatile organic compounds

## Abstract

Wasps, from the genus *Trissolcus,* are egg parasitoids that are commonly used in biological control programs targeting stink bugs. They navigate a complex environment, relying on a diverse array of biochemical and ecological cues to locate their hosts. Through this endeavour, these parasitoid wasps have to discriminate between young and old eggs as development is only achieved in the latter. In this study, we evaluated the ability of two parasitoid wasps, *Trissolcus japonicus* and *Trissolcus basalis,* on utilising short-range cues and, more specifically, volatile organic compounds emitted by stink bug egg masses to locate their hosts. We hypothesised that (1) stink bug eggs (i.e., *Halyomorpha halys* and *Nezara viridula*) emit short-range cues that are exploited by egg parasitoids (i.e., *T. japonicus* and *T. basalis*) to locate their hosts in addition to insect chemical footprints; (2) *Trissolcus* spp. Have the ability to differentiate young eggs from older ones to increase their fitness (3) based on changes in the chemical profiles of the egg masses according to their age. Our behavioural assays suggested that *T. japonicus* did not respond to stink bug footprints, whereas *T. basalis* was significantly oriented toward the footprints of gravid host females. Both parasitoids preferentially oriented towards young eggs rather than footprints. The parasitism rate of *T. japonicus* was not significantly different between young and old eggs unlike *T. basalis* which preferred parasitising on young eggs. We identified γ-butyrolactone and β-funebrene in the headspace of *N. viridula* eggs and we discussed the putative role of these secondary metabolites on *T. basalis locating their host.* Behavioural, performance and VOCs collection of this study contribute to a nuanced understanding of host–parasitoid dynamics along with implications for developing effective pest management strategies.

## Introduction

Herbivorous stink bugs (Hemiptera: Pentatomidae) are pervasive pests threatening diverse agricultural crops, orchards, and wild plants globally (Tillman et al., 2010; Rice et al., 2014; Conti et al., 2021). The primary damage that they cause comes from their piercing and sucking feeding behaviour on fruits and seeds, leading to deformities and discolorations, rendering the final products unsuitable for the market (Leskey et al., 2012). Among this pest complex, *Halyomorpha halys* (Stål) (Hemiptera: Pentatomidae) (brown marmorated stink bug, BMSB) has recently drawn attention from researchers worldwide. *Halyomorpha halys* is a polyphagous species native to Asia (Lee et al., 2013) that has been introduced to the United States (Hoebeke and Carter, 2003), South America (Faúndez and Rider, 2017), and Europe (Wermelinger et al., 2008; Maistrello et al., 2016), where it has become a significant pest (Leskey et al., 2012; Haye and Weber, 2017). *Nezara viridula* (L.) (Hemiptera: Pentatomidae) is native to East Africa and has spread worldwide to become a cosmopolitan polyphagous pest (Todd, 1989; Panizzi and Lucini, 2016).

Currently, management of both species heavily relies on synthetic insecticides, which have significant environmental impacts (Nielsen et al., 2008), including detrimental effects on natural enemies and pollinators (Salerno et al., 2002; Desneux et al., 2007). Therefore, alternative strategies are of utmost importance for the effective and sustainable management of stink bug populations. Among differing alternatives, biological control has shown great potential (Lee, 2015; Conti et al., 2021). In particular, biological control strategies offer a nature-positive alternative to chemical pest control methods. In order to reduce our reliance on pesticides, which are increasingly being withdrawn from the market, it is crucial to bolster biological control measures by fostering the growth of natural enemy communities. This approach can help to restore ecological balance and enhance resilience against pest invasions (Díaz et al., 2019). Among the array of natural enemies of stink bugs, egg parasitoids stand out as particularly effective. Their use in stink bug pest management have thereby prevented nymph and adult damage (Conti and Colazza, 2012).

In the native habitats of *H. halys*, the samurai wasp *Trissolcus japonicus* (Ashmead) (Hymenoptera: Scelionidae) has been identified as the predominant species parasitizing *H. halys* eggs (Kamiyama et al., 2022). *Trissolcus japonicus* can complete multiple generations per year (Qiu et al., 2007; Yang, 2009) and achieve high parasitism rates on *H. halys* eggs in China up to 90%, greater longevity and a higher net reproductive rate than related parasitoid species like *Trissolcus mitsukurii* (Mele et al., 2024). A second parasitoid wasp from the same genus, *T. basalis* (Wollaston) (Hymenoptera: Scelionidae), is frequently used as a biological control agent against *N. viridula* (Jones, 1995; Esquivel et al., 2018). Parasitic wasps navigate a complex environment, relying on a diverse array of biochemical and ecological cues to locate and identify their hosts (Godfray, 1994; Colazza et al., 2004). In addition to visual signals, parasitoid wasps use semiochemicals (Vinson, 1998) to facilitate host location and acceptance. More specifically, egg parasitoid wasps initially exploit herbivore-induced plant volatiles (Vet and Dicke, 1992) pheromones (Fatouros et al., 2008) and kairomones as long-distance cues (Mattiacci et al., 1993). Subsequently, they rely on short-range cues released by host eggs, adding a layer of complexity to the host location process (Vet and Dicke, 1992).

Research on host recognition in *Trissolcus* species highlights the key role of chemical cues emitted by stink bug eggs (Michereff et al., 2016; Tognon et al., 2016; Tognon et al., 2017). However, these cues do not precisely indicate egg location; instead, they attract female parasitoids nearby (Colazza et al., 2007). This nuance adds depth to our understanding of host–parasitoid interaction dynamics. Once the host is located, females of *T. japonicus* and *T*. *basalis* both probe the eggs with the ovipositor before and after parasitisation (Botch and Delfosse, 2018; Salerno et al., 2006). Furthermore, *T. japonicus* and *T. basalis* must discriminate between young and old host eggs as parasitism rates decrease with increasing host egg age (Sabbatini-Peverieri et al., 2020). They have, therefore, evolved the ability to distinguish between freshly laid and older stink bug eggs, as is the case with another species of the family Scelionidae, *Telenomus remus*. This optimises their parasitic behaviour for reproductive success. (Peñaflor et al., 2012).

Egg compounds are indeed an important host-recognition indicator, providing cues of host quality and previous parasitisation (Li et al., 2020). To our knowledge, the chemical mechanisms leading to *T. japonicus* and *T. basalis* oviposition still remain unknown. For these reasons, we assumed that *T*. *japonicus* and *T*. *basalis* may rely on short-range cues derived from host egg surfaces to optimize oviposition site selection. We expected that these cues enable parasitoids to discriminate between viable and non-viable eggs, enhancing reproductive success and biocontrol efficiency by minimizing energy expenditure on unsuitable hosts.

In an effort to unravel aspects of these mechanisms, we hypothesised that.

(1) *Trissolcus japonicus* and *T. basalis* utilize chemical cues from footprints and/or eggs of their respective hosts, *H. halys* and *N. viridula*;
(2) *Trissolcus* spp. Have the ability to discriminate young eggs from older ones to increase their fitness;
(3) VOCs from H. halys and N. viridula egg masses change over their development time.

## Materials and methods

### Stink bug rearing and handling

*Halyomorpha halys* and *N. viridula* adults were collected in apple orchards around Nyon, Switzerland. The two species were reared separately. In the laboratory, the adults were maintained in a plastic box (32.5 × 21.5 × 9 cm). Insects were offered water and a diet—mixture of 3 g (1:1:1) of peeled sunflower seeds, chopped peanuts (Vita-Balance, Landi, Switzerland), and green beans (var. Coco, Migros, Suisse)—as needed. To promote oviposition, three tissue sheets were arranged in a fan-like configuration at the bottom of the box, serving as the oviposition substrate. The use of tissue sheets was selected to prevent the influence of plant odour on the egg masses. Adults were maintained in a climate chamber (Schaub, Geneva, Switzerland) at 24°C, 65% RH, and under a photoperiod of 16:8 h L:D. Eggs were collected daily directly from the rearing cage. Based on the oviposition date and egg development (Rice et al., 2014), eggs were classified as young (1–3 days old) and old (4–6 days old). The collected eggs were placed in plastic boxes (19.5 × 13.5 × 5 cm) and stored in a climate chamber at 20°C and 70% RH with a photoperiod of 16:8 h L:D.

### Parasitoid rearing

Laboratory colonies of *T. japonicus* and *T. basalis* (Andermatt, Switzerland) were established on freshly laid *H. halys* and *N. viridula* eggs, respectively. Emphasis was placed on utilising fresh eggs; however, if this resource was depleted, frozen eggs (kept at −80°C) of the respective host species were substituted (McIntosh et al., 2019). The parasitoids were reared in cages (61 × 33 × 41 cm, Nhbs, Bonn, Germany). In addition to water, three blotting papers coated with honey and sprinkled with pollen provided a suitable nutritional source for the parasitoids. Egg masses for parasitism were strategically placed at the centre of the cage to facilitate natural behaviour. All cages were maintained under controlled conditions in a climatised room (24°C ± 1°C, 70% RH, and a photoperiod of 16:8 h L:D). The rearing cages were distinguished according to the use of parasitoids. In all behavioral bioassays, native parasitoids no older than 7 days were used. These individuals were considered naïve, as they had not been previously exposed to host eggs. Parasitoids emerged directly from host eggs under controlled rearing conditions, ensuring they had no prior parasitic experience.

### Behaviour–Footprint assay

**FIGURE 1:**
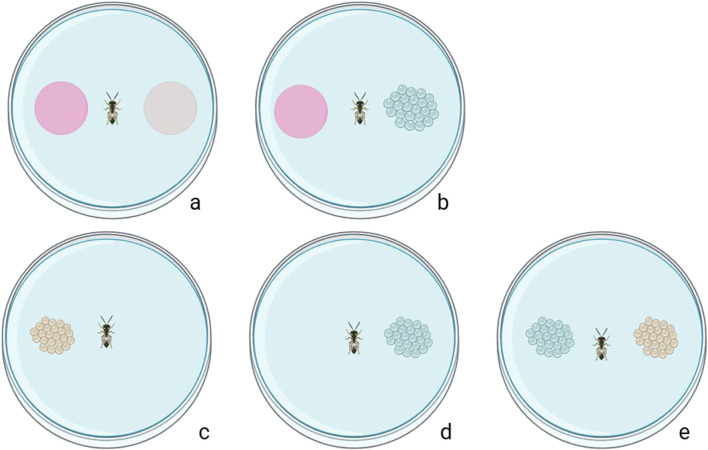
Schematic representation of the footprint-assay and performance assays: **(a)** choice test of *Trissolcus* females between footprint and control (filter paper only); **(b)** choice test of *Trissolcus* females between footprint and young eggs; **(c)** performance with old eggs only; dperformance with young eggs; **(e)** performance with young versus old eggs).

Gravid females of *H. halys* and *N. viridula* were introduced into a 60 × 15 mm Petri dish containing a 50 mm diameter filter paper disc. Dishes were then set aside for 1 h at room temperature allowing insects to deposit footprints onto the filter paper disc. Control discs were prepared following the same procedure but without exposure to insects. The assay comprised of two distinct combinations for each parasitoid species: (a) young egg masses versus gravid female footprints (b) gravid female footprints versus control (Figure 1). In this assay, parasitoids were exposed exclusively to the footprints or eggs of their respective host species. Naïve *T. japonicus* and *T. basalis* of 7 days old, were then individually placed in the centre of glass Petri dishes (120 × 20 mm). The parasitoid behaviour was observed after 15 min, 30 min, and 1 h. Choices were confirmed if females oviposited on young eggs or were located on the filter paper; otherwise, individuals were considered non-responding. After 1 h, the females and egg masses were removed from the arenas and excluded from subsequent tests. A total of 40 replicates per combination were conducted at room temperature. Petri dishes were cleaned after each replicate with acetone and pentane prior to being sterilized for 2 h at 200°C.

### Behaviour–Y-olfactometer assays

**FIGURE 2:**
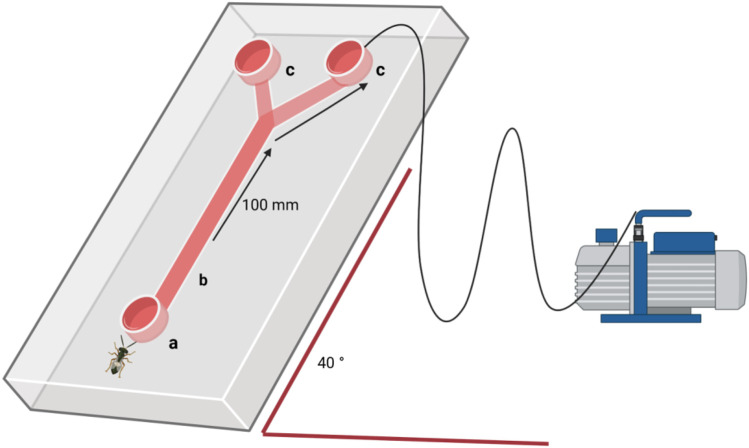
Schematic representation of an olfactometer assay at 40° angle, connected to a vacuum pump: **(a)** parasitoid entrance; **(b)** neutral zone; **(c)** choice zone with egg masses.

Behavioural responses of *T. japonicus* and *T. basalis* were observed in a Y-olfactometer (Figure 2, stem: 100 mm, arms: 100 mm at a 30° angle between arms, internal diameter: 20 mm, outside diameter: 22 mm). The olfactometer was positioned at a 40° angle in relation to the substrate for optimal parasitoid response. Airflow (0.25 L min^-1^) was maintained constant through the olfactometer arms with an air delivery system (Clean Air Supply System CASS6, VAS, NY, United States) with charcoal filters (Figure 2). The reared parasitoids were at most 7 days old, and defined as naïves, as they had never been exposed to the eggs before the behavioural tests. The wasps were given a choice between the young and old eggs of their respective hosts, with equal egg numbers in each mass. Responses were observed after 15 min, 30 min, and 1 h. The time intervals of 15 and 30 min were added to help determine whether the parasitoid changed its choice before making a final decision. All time intervals were considered in the data analysis for each parasitoid. The stem of the olfactometer was considered the neutral zone, and parasitoids retrieved from this zone were classified as non-responding. Two olfactometers were used simultaneously. Females entering at least 4 cm into the test arm was counted as a choice. After 1 h, the females and egg masses were removed, and the olfactometer was cleaned with acetone and pentane and oven-sterilized at 200°C for 2 h between replicates. A total of 40 replicates per species were conducted.

### Performance–No choice assays

Mated females of *T. japonicus* and *T. basalis* were individually placed in the centre of 60 × 15 mm Petri dishes. Each arena contained either a young or an old egg mass (Figure 1). The position of the tested parasitoid in the experimental area was recorded after 15 min, 1 h, and 24 h. A choice was confirmed when the female oviposited in the eggs (visual check on a microscope); otherwise, it was considered non-responding. After 24 h, the females and egg masses were removed, and the number of emerging nymphs, unhatched eggs, parasitism rate, and sex ratio of emerged parasitoids were recorded. A total of 40 replicates for both young and old egg masses were conducted in a climatised room (24°C ± 1°C, 70% RH, and a photoperiod of 16:8 h L:D).

### Performance–Dual-choice assays

In the dual-choice behavioural experiment, a 7 day old single-mated *T. japonicus* or *T. basalis* female was placed in a small arena (90 mm Petri dish) with two egg clusters, with each egg cluster having an equal number of 26 eggs, of both ages (young and old) of their respective host (Figure 1). The position of the parasitoid in the arena was observed after 15 min, 1 h, and 24 h, considering oviposition as the confirmation of host selection. After 24 h, the female and egg masses were removed, and the number of emerging nymphs, unhatched eggs, parasitism rate, and sex ratio of the emerged parasitoids were recorded. A total of 40 replicates were conducted in a climatised room (24°C ± 1°C, 70% RH, and a photoperiod of 16:8 h L:D).

### Egg volatile organic compounds collection and analysis

Given the difficulty of collecting egg-emitted volatile organic compounds (VOCs) without sample destruction, we opted to extract VOCs from grounded frozen eggs with solid-phase microextraction (SPME) fibres. This step aimed to enhance the efficiency of VOC extraction by providing better access to compounds in the headspace and by exploring the internal compounds of the egg masses. Additionally, increased surface area resulting from sample fragmentation improved adsorption onto the SPME fibre (Curran et al., 2016; Gaikwad et al., 2022). Notably, our study avoided the use of solvents during the collection of egg-emitted VOCs, as described in Michereff et al. (2016) and Tognon et al. (2016). Young and old egg masses of *N. viridula* and *H. halys* were individually deep-frozen in liquid nitrogen and cryo-grounded into a fine powder. The number of eggs per mass for *N. viridula* was standardised to 70–100 eggs, while those of *H. halys* were standardised to 20–28 eggs. Aliquots of the resulting powder (approx. 300 mg for *N. viridula*; approx. 100 mg for *H. halys*) were placed in glass vials (20 mL, Gerstel, Germany) and sealed with a PTFE septum. The exact weight of each sample was recorded for further standardisation. Samples were stored at −80°C prior to analysis.

Vials were submerged in a water bath maintained at 30 ± 1°C for 3 h to optimise the release of VOCs during sampling. VOCs were then collected on a 50/30 μm DVB/CAR/PDMS fibre (Supelco, Bellefonte, PA, United States) previously prepared at 250°C for 1 h. Six replicates per insect species were performed along with two control samples (empty glass vials). Six control samples were prepared by conducting SPME collections on empty vials.

VOC analyses were conducted in a 7890B gas chromatograph (GC) (Agilent Technologies, United States) coupled with a 7,010 triple quadrupole mass spectrometer (MS) (Agilent Technologies, United States). The SPME fibre was conditioned for 30 min at 250°C before use. VOCs were then injected (splitless mode) on a DB-5 capillary column (30 m × 0.25 mm I.D, 0.25 μm film thickness, Agilent J&W, Santa Clara, CA, United States) using helium as the carrier gas at a constant flow of 1.2 mL min^−1^. The GC oven temperature was kept at 50°C for 3 min and then ramped up to 180°C at 5°C min^−1^ This was then followed by a final ramp up of temperature at 8°C min^−1^ to achieve 250°C. This final temperature was held for 3 min. The MS analyses were performed with electron impact ionisation at 70 eV and the ion source was set at 230°C. Data acquisition was conducted in scan mode in the mass range of 35–350 amu. Mass spectra were compared with the Nist14.L library and pure standards were injected for confirmation. The Kovats retention index was calculated for each compound using linear alkanes (C4–C24) and semi-quantitative analysis of butyrolactone was based on the injection of a standard diluted in hexane. The relative concentrations of the isolated volatiles were standardised by the individual sample weight.

### Statistical analyses

All statistical analyses were conducted using R (version 2025.05.0-496).

The evaluation of parasitoid choices, in double choice, footprint and olfactometer assays, was analysed by a generalized linear model (GLM) with a binomial distribution and a logit link function and was used to assess the effect of the interaction between observation time (15 min, 30 min, 1 h or 24 h) and modality (control or footprints; eggs or footprints; young or old eggs) on the choice behavior of *T*. *japonicus* and *T. basalis* (binary response: 1 = choice, 0 = no choice). A combined factor variable (timemodality) was created from the “time” and “modality” factors to model their interaction. A *post hoc* comparison was conducted using the emmeans package, with a significance threshold of *p* ≤ 0.05. The model was fitted with 239 degrees of freedom for the null model and 234 residual degrees of freedom for residuals.

In the performance tests conducted in Petri dishes (no choice and double choice tests), generalized linear models (GLMs) with a Poisson distribution and a log link function were used to analyze the effect of egg age on three response variables: the number of emerged nymphs, the number of emerged parasitoids, and unhatched egg. An offset term based on the total number of eggs (log (tot_eggs)) was included in each model to correct for differences in sample size. The predictor variable “age” was treated as a factor. A *post hoc* comparison was conducted using the emmeans package, with a significance threshold of *p* ≤ 0.05. The model was fitted with 79 degrees of freedom for the null model and 78 residual degrees of freedom for residuals.

VOC peak areas were compared between old and young eggs for each stink bug species using one-way ANOVA and the Kruskal–Wallis test (*p* ≤ 0.05).

## Results

### Behaviour–Footprint assay

**FIGURE 3:**
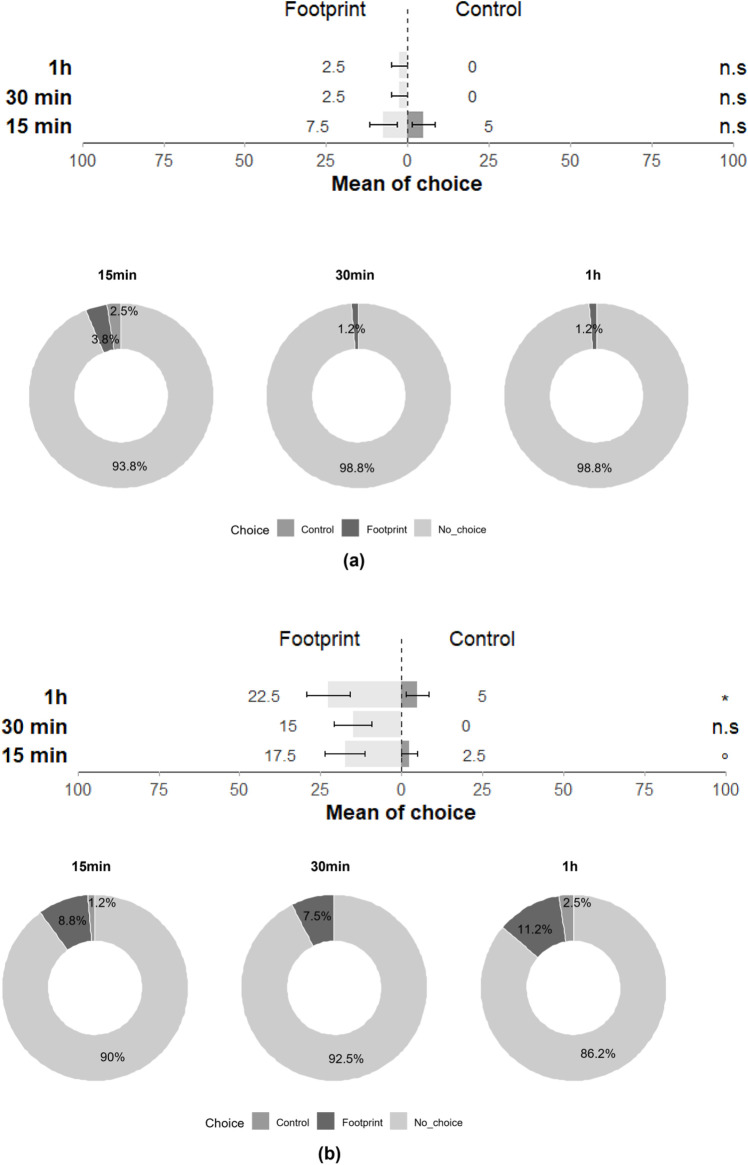
Choice of **(a)** *T. japonicus* and **(b)** *T. basalis* between the filter paper with the footprints of a gravid female of its host and the control filter paper in a glass Petri dish at 15 min, 30 min and 1 h (40 replicates for each species). Stars *** indicate statistical difference (*p* ≤ 0.001) and ° marginally significant difference (0.08 ≤ p < 0.05). n.s states for non-significant difference.

The response of *T. japonicus* and *T. basalis* to the footprints of gravid stink bug females was investigated. Interestingly, *T. japonicus* females did not exhibit attraction towards *H. halys* footprints (15 min: Z = −0.459; *p* = 0.65; 30 min: Z = −0.006; *p* = 0.99; 1 h: Z = −0.006; *p* = 0.99), with only 15% of individuals responding to the cues at least once across the three-time intervals and 85% showing no response (Figure 3). Conversely, *T. basalis* females displayed significant chemotaxis towards the footprints of gravid *N. viridula* females at 1 h and nearly had as much preference for the footprints at 15 min (15 min: Z = −1.930; *p* = 0.054; 30 min: Z = --0.016; *p* = 0.99; 1 h: Z = −2.087; *p* = 0.04), with 50% of individuals responding positively at least once across the three-time intervals (Figure 3).

**FIGURE 4:**
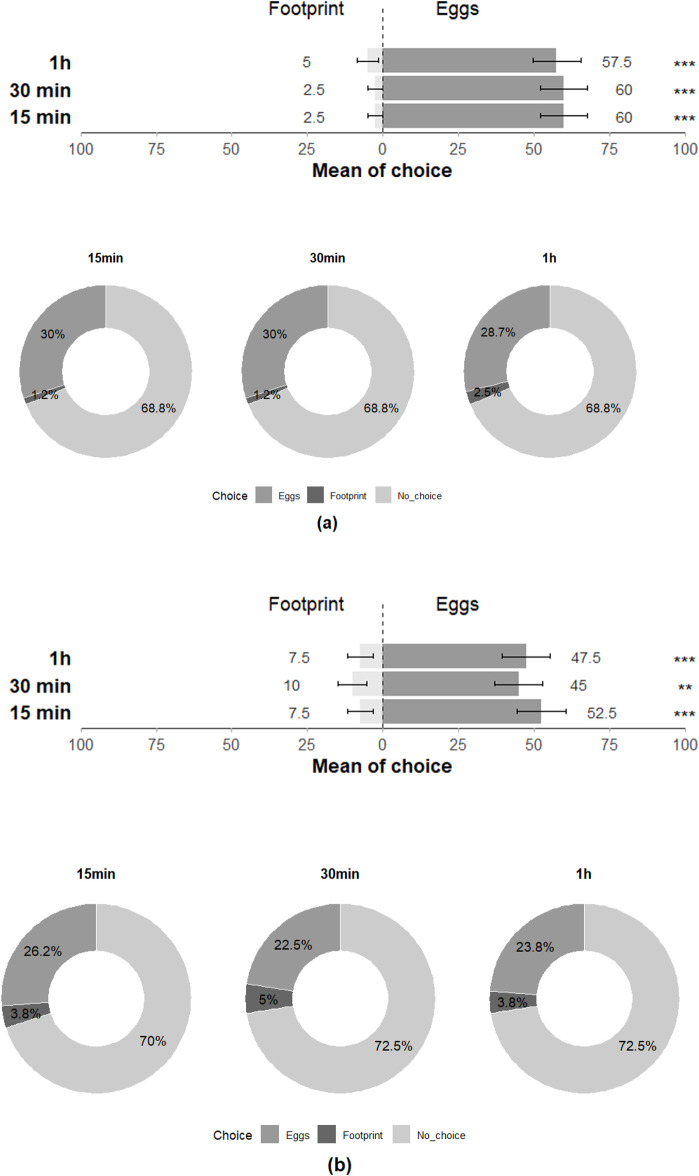
Choice of **(a)** *T. japonicus* and **(b)** *T. basalis* between young eggs from their host and the filter paper with the footprints of a gravid female of its host in a glass Petri dish at 15 min, 30 min and 1 h (40 replicates for each species). *** indicate a statistical difference (*p* ≤ 0.001).

In the double-choice assay, where parasitoids were presented with a choice between young eggs and footprints, a significant orientation towards young eggs was observed for *T. japonicus* for all three-time intervals (15 min: Z = −3.828; *p* < 0.001; 30 min: Z = −3.828; *p* < 0.001; 24 h: Z = −4.095; *p* < 0.001), with 77.5% of individuals responding at least once across the three time intervals and 22.5% showing no response (Figure 4). Similarly, *T. basalis* exhibited a significant preference for young eggs for all three-time intervals (15 min: Z = −3.849; *p* < 0.001; 30 min: Z = −3.244; *p* < 0.01; 24 h: Z = −3.554; *p* < 0.001), with 67.5% of individuals responding at least once across the three-time intervals (Figure 4).

### Behaviour–Y-olfactometer assays

**FIGURE 5:**
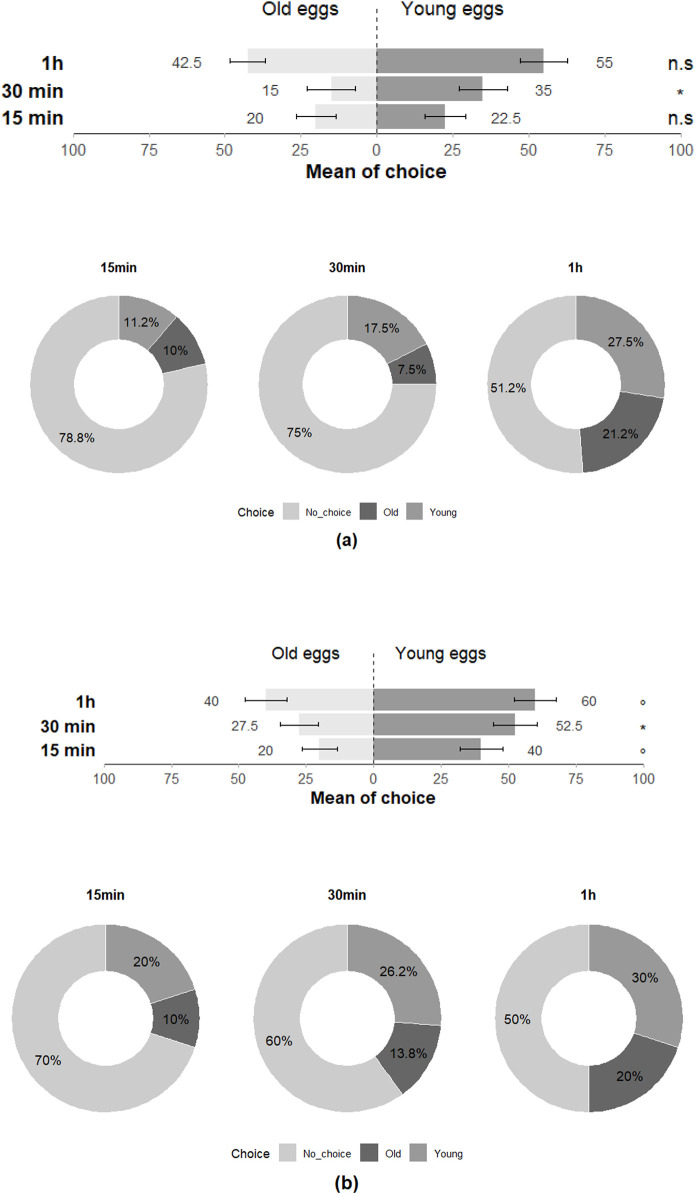
Choice of **(a)** *T. japonicus* and **(b)** *T. basalis* between young and old eggs of their host in olfactometer tests at 15 min, 30 min and 1 h (40 replicates for each species). Stars * indicate statistical difference (*p* ≤ 0.05) and ° marginally significant difference (0.08 ≤ p < 0.05). n.s states for non-significant difference.

In the Y-olfactometer, *T. japonicus showed* significant choice preference between young and old eggs of *H. halys* at 30 min but showed no discrimination at 15 min and 1 h (15 min: Z = −0.273; *p* = 0.79; 30 min: Z = −2.017; *p* = 0.04; 1 h: Z = −1.115; *p* = 0.27), with all individuals (100%) responding positively to the stimuli at least once across the three time intervals (Figure 5). *Trissolcus basalis* exhibited a significant preference for young eggs over old eggs at 30 min and nearly had as much preference for young eggs at 15 min and 1 h (15 min: Z = −1.922; *p* = 0.055; 30 min: Z = −2.251; *p* = 0.02; 1 h: Z = −1.777; *p* = 0.08), with all individuals (100%) responding positively at least once across the three time intervals and highlighting their preference for the olfactory cues associated with young eggs (Figure 5).

### Performance–No choice assays

**FIGURE 6:**
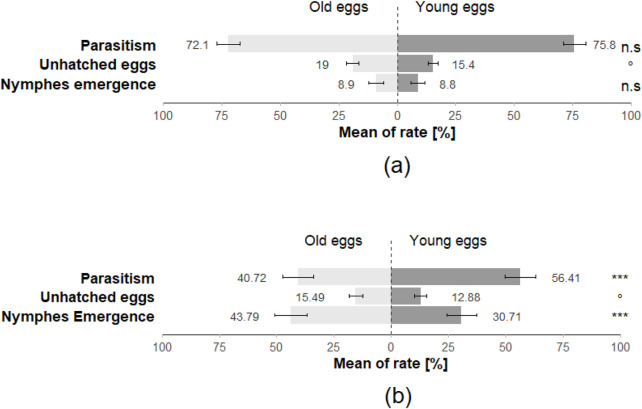
The rate of emergence of stink bugs nymphs, the rate of parasitism and the rate of unhatched eggs for **(a)** *Halyomorpha halys* eggs (parasitised by *T. japonicus*) and for **(b)** *Nezara viridula* eggs (parasitised by *T. basalis*) in the no choice arena trials (40 replicates for each species). Stars *** indicate statistical difference (*p* ≤ 0.001) and stars * indicate statistical difference (*p* ≤ 0.05) and marginally significant difference (0.08 ≤ p < 0.05). n.s. Indicates non-significant difference.

Regarding the performance of *T. japonicus* on young and old *H. halys* eggs, the emergence rate of nymphs was nearly 9% in both cases. The parasitism rate for young eggs was 75%, contrasting with 71% for old eggs. Furthermore, the rate of unhatched eggs was 16% for young eggs, whereas it rose to 20% for old eggs (Figure 6; Table 1). There was no significant difference in the emergence rate of nymphs after parasitisation (Z = 0.132; *p* = 0.89) and in the parasitism rate (Z = 0.906; *p* = 0.365). However, egg age had a marginal impact on the rate of unhatched eggs (Z = −1.919; *p* = 0.055) (Figure 6; Table 1).

**TABLE 1:** Summary of mean values (±SE) for total eggs used, emerged nymphs, emerged parasitoids (with sex-ratio), and unhatched eggs by age and species in no-choice arena tests (40 egg masses for each species and age).

	*Halyomorpha halys*		*Nezara viridula*	
	Young eggs	Old eggs	Young eggs	Old eggs
Eggs	23.7 ± 0.651	24.2 ± 0.697	70.1 ± 3.17	82.6 ± 2.7
Emerged nymphes	2.22 ± 0.795	2.22 ± 0.755	25 ± 5.4	37.1 ± 6.15
Emerged parasitoids	17.7 ± 1.13	17.2 ± 1.22	35.4 ± 4.22	32.7 ± 5.38
Parasitoids sex-ratio (females: male)	7.22:1	10.08:1	10.08:1	7.77:1
Unhatched eggs	3.8 ± 0.523	4.78 ± 0.722	9.6 ± 1.85	12.8 ± 2.38

Conversely, the performance of *T. basalis* on *N. viridula* eggs revealed notable differences. For young *N. viridula* eggs, the hatching rate of nymphs stood at 36%, while increasing to 45% for old eggs. The parasitism rate for young eggs was 50%, in contrast to 40% for old eggs. Additionally, the rate of unhatched eggs was 14% for young eggs, whereas it rose slightly to 15% for old eggs (Figure 6). The statistical analysis of *N. viridula* data indicated a highly significant difference in the emergency rate of nymphs (Z = −5.599; *p* < 0.001) and the overall rate of parasitism (Z = 6.422; *p* < 0.001). Moreover, the rate of unhatched eggs showed a marginal difference (Z = −1.88; *p* = 0.06) (Figure 6; Table 2).

**TABLE 2:** Summary of mean values (±SE) for total eggs used, emerged nymphs, emerged parasitoids (with sex-ratio), and unhatched eggs by age and speciesin double-choice arena tests (40 egg masses for each species and age).

	*Halyomorpha halys*		*Nezara viridula*	
	Young eggs	Old eggs	Young eggs	Old eggs
Eggs	23.9 ± 1.06	24.4 ± 0.976	81.2 ± 2.62	81 ± 2.99
Emerged nymphs	8.38 ± 1.29	9.57 ± 1.35	34.4 ± 5.72	39.6 ± 5.04
Emerged parasitoids	7.58 ± 1.31	7.32 ± 1.37	30.5 ± 4.32	24 ± 4.76
Parasitoids sex-ratio (females: male)	5.73:1	3.13:1	7.58:1	5.67:1
Unhatched eggs	7.92 ± 1.01	7.55 ± 0.98	16.3 ± 2.47	17.4 ± 2.18

### Performance–Double-choice assays

**FIGURE 7:**
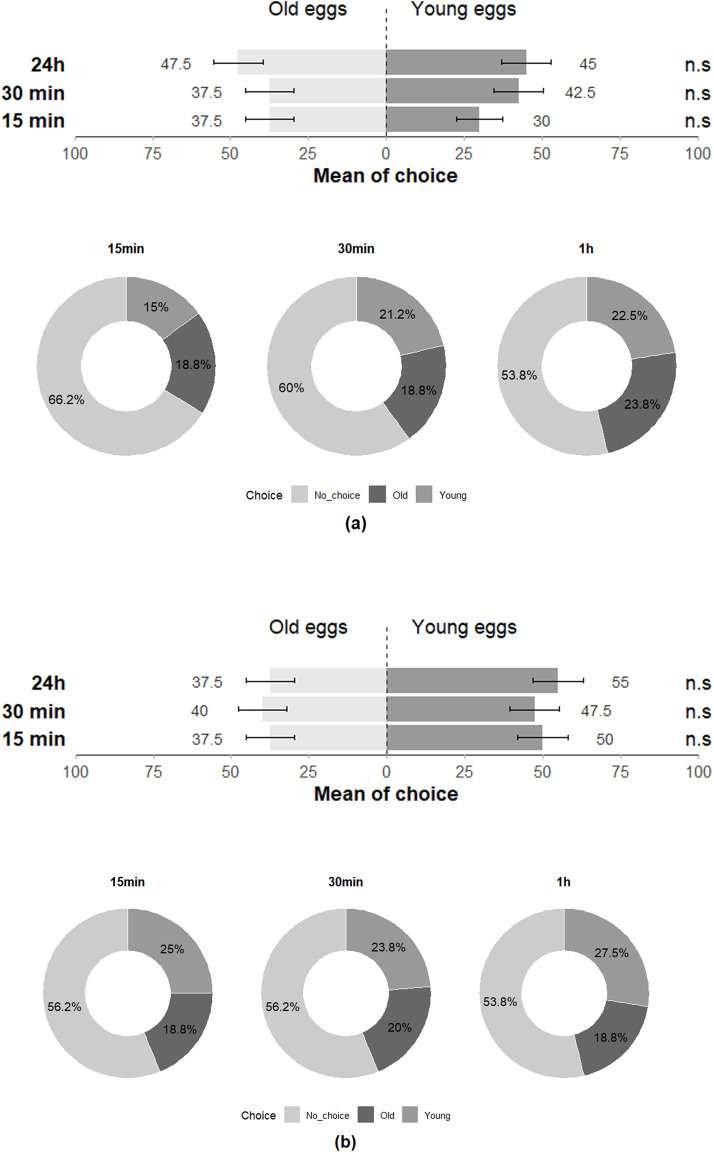
Choice of **(a)** *T. japonicus* and **(b)** *T. basalis* between young and old eggs of their host in the double-choice arena trials at 15 min, 30 min and 24 h (40 replicates for each species). n.s states for non-significant difference.

**FIGURE 8:**
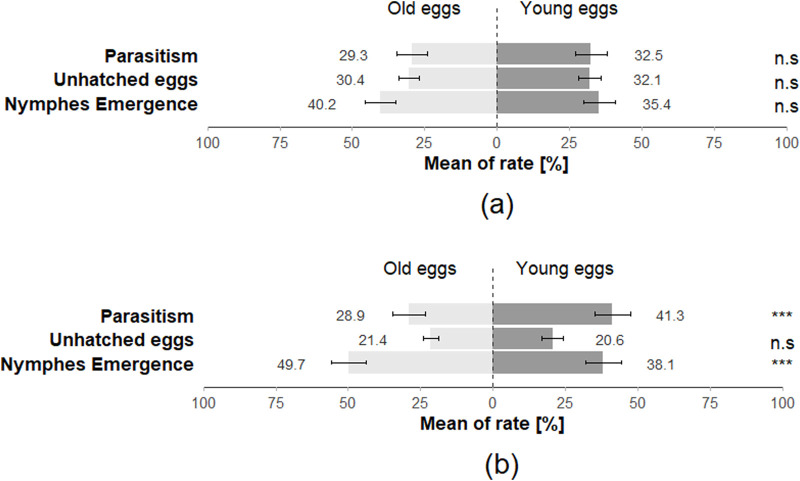
The rate of emergence of stink bugs nymphs, the rate of parasitism and the rate of unhatched eggs for **(a)** *Halyomorpha halys* eggs (parasitised by *T. japonicus*) and **(b)** *Nezara viridula* eggs (parasitised by *T. basalis*) in the double-choice arena trials (40 replicates for each species). Stars *** indicate statistical difference (*p* ≤ 0.001) and ° marginally significant differences. n.s. Indicates non-significant difference.

The preference of *T. japonicus* between young and old eggs of its host species also showed a non-significant difference for all three-time intervals (15 min: Z = 0.708; *p* = 0.48; 30 min: Z = −0.456; *p* = 0.65; 24 h: Z = 0.224; *p* = 0.82), with 97.5% of individuals making a choice at least once across the three-time intervals (Figure 7). The performance of *T. japonicus* on *H. halys eggs* exhibited no significant difference in the rate of nymph emergence (Z = −1.472; *p* = 0.1), in parasitism rate (Z = 0.7; *p* = 0.4) and rate of unhatched eggs (Z = 0.899; *p* = 0.4) (Figure 8; Table 2).

Similar to the *H. halys-T. japonicus* performance, the choice made by *T. basalis* between the young and old eggs of its host demonstrated a non-significant difference for all three-time intervals (15 min: Z = −1.124; *p* = 0.3; 30 min: Z = −0.675; *p* = 0.5; 24 h: Z = −1.561; *p* = 0.1), with 97.5% of individuals responding positively at least once across the three time intervals (Figure 7). However, the emergence rate of *N. viridula* nymphs was affected by the age of the egg mass exposed to *T. basalis* revealing a highly significant difference between young and old eggs in the emergence rate of stink bug nymphs (Z = 3.884; *p* < 0.001) and for the parasitism rate (Z = −5.478; *p* < 0.001), while the rate of unhatched eggs showed a non-significant difference (Z = 1.217; *p* = 0.2) (Figure 8).

### Identification of VOCs

**FIGURE 9:**
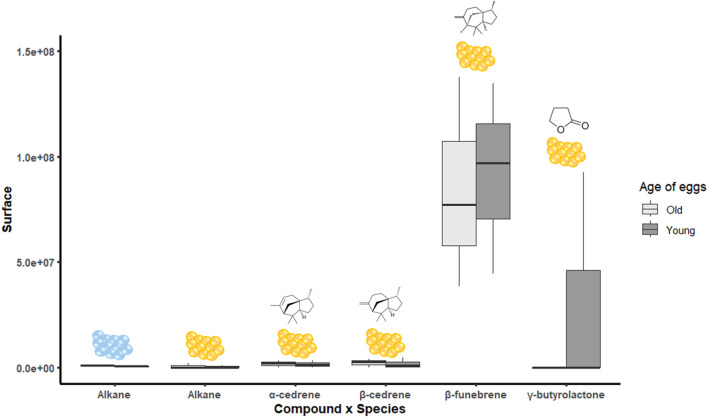
Peak area of each compound identified in old or young eggs of *Halyomorpha halys* (blue eggs) and *Nezara viridula* (yellow eggs). Absence of letters means no statistical difference.

We identified α-cedrene (mean RI = 1,271), β-cedrene (mean RI = 1,372.5), β-funebrene (mean RI = 1,346.5), and γ-butyrolactone (RI = 1,401) in *N. viridula* egg masses. A C13 alkane (mean RI = 1,102.8) was found in both H. *halys* and *N. viridula* eggs but could not be identified based on available libraries. Except for the C13 alkane, no other compound was isolated from *H. halys* egg masses. No significant differences were observed between old and young *N. viridula* eggs due to high variability (Figure 9), where *N. viridula* old egg masses tended to release more γ-butyrolactone. The semi-quantitative assessment showed that γ-butyrolactone is present in young *N*. *viridula* eggs at levels of 10.14 ng/g fresh tissue, as determined by the molecular ion (m/z = 86), and 18.69 ng/g fresh tissue, based on the base peak (m/z = 42).

## Discussion

### Parasitoid behaviour

Our findings reveal that both parasitoid species*, T. japonicus* and *T. basalis*, exhibited a preference for young over older eggs. From an ecological perspective, younger eggs are more susceptible to successful parasitisation as they provide an opportunity for a longer development time for the parasitoid and as parasitoids do not compete with stink bug nymphs that are already in the development stage. This increases the parasitoids’ chances of survival and successful emergence. Based on our results, we can conclude that parasitoids showed a stronger preference for young eggs when only volatile cues were available suggesting that olfaction plays a major role in this foraging behaviour.

In the arena experiments, when both footprints and young eggs were simultaneously offered, *T. basalis* strongly preferred eggs. The parasitoids selected the egg masses within an hour and did not change their choice (Figures 3 and 4). This suggests that eggs have an olfactory importance in the parasitoids’ host-seeking behaviour. However, we cannot exclude the possibility that clear host-foraging behaviours were not observed (e.g., brief motionless periods with full antennal contact with the substrate, decreased walking velocity, and increased turning velocity) (Colazza et al., 1999; Colazza et al., 2007; Peri et al., 2016).

Confirming this behavioural preference, the parasitism rate of young eggs was higher and the respective emergence rate of parasitoid wasps followed the same trend. In line with the preference–performance hypothesis, these results suggest that *T. japonicus* and *T. basalis* employ short-range cues from eggs to locate their oviposition sites. Moreover, following the mother knows best theory (Scheirs et al., 2000; Mayhew, 2001), *T. japonicus* and *T. basalis* assess the quality of their hosts to ensure optimal conditions for their progeny. A recent study from Tian et al. (2025) showed that *Trichogramma japonicum* (Hymenoptera: Trichogrammatidae) females were attracted to the best quality host eggs exploiting their odours. Our findings may offer mechanistic insights that complement a previous study by Qiu et al. (2007), which demonstrated a preference for young eggs without exploring the role of VOCs. The outcomes of our choice tests align with the behaviour of other *Trissolcus* species, such as *Trissolcus semistriatus* (Nees) and *Trissolcus megallocephalus* (Ashmead), both of which exhibited a preference for young eggs of their hosts over old ones (Awadalla, 1996; Kivan and Kilic, 2004). All female parasitoids used in our behavioural tests were naïve, with no prior parasitism experience. Experience is an important factor of egg parasitoids with indirect host-related cues being able to lead to behavioural responses (Salerno et al., 2009; Peri et al., 2016). However, the oviposition experience is also an important factor to consider in VOCs blend recognition. For example, D’Alessandro and Turlings (2005) showed that compared to naïve wasps, experienced wasps of *Cotesia marginiventris* (Hymenoptera: Braconidae) were more strongly attracted to a specific blend of maize seedlings after the blend was discerned while ovipositing. Oviposition experience plays a critical role in shaping the behaviour and reproductive strategies of naïve parasitoids. For example, in *Anastatus japonicus* (Hymenoptera: Eupelmidae) experienced females lay more eggs per clutch and exhibit enhanced reproductive efficiency (Wang et al., 2023).

It is essential to note that our experiments were conducted with eggs on artificial substrates. Previous studies on *T. basalis* indicated that attraction to eggs was more effective when the plant had been damaged by stink bugs (Colazza et al., 2004), underscoring the importance of additional cues and more complex VOC blends in the oviposition behaviour of *Trissolcus* species. Consequently, two hypotheses can be derived from these data: (a) *T. japonicus* may need more specific stimuli to recognise its host which,for example, may require more natural interactions (e.g., eggs laid directly on the leaf) to distinguish its host more specifically and consequently enhance parasitism, or (b) the olfactory profile of *H. halys* eggs is less diversified, making host recognition more challenging for *T. japonicus*.

### Eggs-emitted VOCs

In this study, our comprehensive VOC collection facilitated the detection of compounds encompassing alkanes, sesquiterpenes, and lactones in the eggs of *H. halys* and *N. viridula*. Although a C13 alkane was isolated in both species, its identification remains elusive, whereas other alkanes have already been identified in *H. halys* and have been found to be often used as a defence metabolite (Weber et al., 2017). Malek et al. (2021) reported the presence of (*E*)-2-decenal and n-tridecane in *H. halys* nymphal instars and adults with a higher abundance in male *H. halys* extracts. Behavioural tests conducted on female *T. japonicus* revealed that n-tridecane led to a longer residence time on the filter paper compared to (*E*)-2-decenal. The blend of both compounds produced similar results to n-tridecane alone. Zhong et al. (2017) supported these findings by indicating that *T. japonicus* females were attracted to n-tridecane and repelled by (*E*)-2-decenal. These defensive compounds are typically emitted by the dorsal abdominal glands of nymphs and the metathoracic glands of adults (Lopes et al., 2015). To the best of our knowledge, this is the first time that alkanes have been isolated from Pentatomidae egg masses suggesting that this chemical defence is conserved at this developmental stage. Understanding whether these alkanes, likely transmitted by the mother, play a defensive role against natural egg enemies remains to be elucidated. No significant difference was observed between young and old eggs’ constitutive alkane profiles and further analyses should be conducted to characterise the actual release of these VOCs in the egg vicinity and potential age-dependent bioactivity.

In addition to alkanes, we identified sesquiterpenes in the egg masses, although exclusively in *N. viridula*. Similarly to alkanes, sesquiterpenes can also play a defensive role against pathogens and natural enemies. The sesquiterpene β-funebrene is not extensively discussed in entomological studies. In addition, sesquiterpenes play a role in parasitoid attraction (Hoballah and Turlings, 2005; D’Alessandro and Turlings, 2005; Ayelo et al., 2022). In our study, the sesquiterpenes α-cedrene, β-cedrene, and β-funebrene have not been individually tested and, to our knowledge, there is no available study related to their role as attractive compounds for parasitoids. However, the isomer α-funebrene is recognised as an oviposition-induced plant volatile produced by *Brassica nigra* L. during oviposition by *Pieris brassicae* L. (Lepidoptera: Pieridae) (Fatouros et al., 2012; Reymond, 2022), and the emission of α-funebrene increases with time. Our data suggests that β-funebrene could also be present on the eggs of certain pentatomids, potentially resulting in the attraction of parasitoids. Further research on these compounds is yet needed to draw conclusions.

The lactone γ-butyrolactone has been detected in *N. viridula* eggs but with significant variability between samples. Further analysis is necessary to identify and quantify all the compounds present in the eggs. This lactone has also been identified in the metathoracic glands of *Aethus indicus* (Westwood) (Hemiptera: Pentatomidae). Lactones in insects often play a defensive role as demonstrated in beetles (e.g., dodecalactone) and bees (e.g., decalactone, decalactone) (Olagbemiro et al., 1984). Since the development of parasitoid offspring decreases as egg age increases (Godfray, 1994), γ-butyrolactone may serve as an indicator of egg quality for females, enabling them to choose eggs that offer the best conditions for successful oviposition. Consequently, γ-butyrolactone may function as a cue that helps females identify and select eggs most suitable for optimal progeny development.

Overall, the collection resulted in a narrow spectrum of compounds. Notably, the eggs were laid on an artificial substrate exclusive of plant influence. However, the development of the embryo in the egg is influenced by the plants’ secondary metabolites, which are incorporated into the eggs and serve as protection from parasitoids and predation (Blum and Hilker, 2003). We also hypothesise that for a survival strategy, there is no ecological interest for the eggs to have a particularly attractive odour, as discretion might be one of the few defence mechanisms. Physiological interactions with the plant could certainly result in the release of more complex compound blends. Therefore, we expect a greater presence of external and internal egg compounds in cases where the egg was laid directly on the plant. The aim of this study was to detect the egg’s odour in the absence of the plant’s influence. Future experiments are necessary to compare the rate of parasitisation between the two types of oviposition.

Based on the literature describing the defensive effect of some compounds herein isolated, we hypothesise that these compounds may defend egg mass from external microbial infections. Despite the low complexity of the blends released by egg masses, parasitoids could still use these VOCs as cues to locate the eggs of their insect hosts. *Trissolcus japonicus* and *T. basalis* can discriminate between young and old eggs, thereby increasing their fitness. Our data supports the hypothesis that chemical cues released by egg masses are used by parasitoids to precisely locate oviposition sites. Nevertheless, this mechanism still needs to be demonstrated through further behavioural tests.

## Data Availability

The raw data supporting the conclusions of this article will be made available by the authors, without undue reservation.
